# Metformin synergistically increases the anticancer effects of lapatinib through induction of apoptosis and modulation of Akt/AMPK pathway in SK-BR3 breast cancer cell line

**DOI:** 10.22038/IJBMS.2021.58825.13069

**Published:** 2021-11

**Authors:** Davood Neamati, Azam Khedri, Mohammad Aberomand, Ali-Asghar Hemmati, Maryam Mohammadzadeh, Kourosh Akbari Baghbani, Ghorban Mohammadzadeh

**Affiliations:** 1 Department of Clinical Biochemistry, School of Medicine, Ahvaz Jundishapur University of Medical Sciences,Ahvaz, Iran; 2 Toxicology Research Center, Department of Clinical Biochemistry, Faculty of Medicine, Ahvaz Jundishapur University of Medical Sciences, Ahvaz, Iran; 3 Medicinal Plant Research Center, Department of Toxicology, School of Pharmacy, Ahvaz Jundishapur University of Medical Sciences, Ahvaz, Iran; 4 Translational Ophthalmology Research Center, Tehran University of Medical Sciences, Tehran, Iran; 5 Department of Infection, Immunity and Inflammation, University of Leicester, LE1 7RH, UK.; 6 Hyperlipidemia Research Center, Department of Clinical Biochemistry, School of Medicine, Ahvaz Jundishapur University of Medical Sciences, Ahvaz, Iran

**Keywords:** Akt, AMPK, Apoptosis, Drug synergism, Lapatinib, Metformin

## Abstract

**Objective(s):**

Combination chemotherapy is a beneficial intervention for breast cancer, versus single therapy. We investigated the effect of Metformin (Met) on Lapatinib (Lap)-induced apoptosis in SK-BR3 cells.

**Materials and Methods:**

Toxic effect of Met and Lap on SK-BR3 cells was measured using MTT assay. Flow cytometry was used to measure the co-treatment effect of Met on lapatinib-induced apoptosis. The relative expression of Bax, Bcl2, and P21 was measured using a real-time PCR. The activity of caspase 3 and 9 was measured using an ELISA kit. The protein level of AMPK and Akt was determined using Western blot analysis.

**Results:**

Metformin and lapatinib alone and combined form showed significant time- and dose-dependent toxic effects on SK-BR3 cell viability. The greatest synergistic inhibitory effect on the cell viability [combination index (CI) = 0.51] was remarkable at Met 100 mM combined with Lap 100 nM. The combination has a stronger apoptotic death (46%) versus lapatinib alone. The combination considerably increased the mRNA expression of Bax and P21, and caspase 3 and 9 activity, while, decreasing the mRNA expression of Bcl2. Additionally, the combination significantly up-regulated and down-regulated the protein levels of AMPK and Akt, respectively.

**Conclusion:**

The metformin-lapatinib combination can induce more potent apoptotic death versus each compound individually. The combination may be suggested as a valuable therapeutic intervention in patients with breast cancer. However, additional in vivo studies are necessary to evaluate the clinical use of the combination for induction of apoptosis and its antitumor effects.

## Introduction

Breast cancer, the most common malignancy in women, nowadays has outstripped lung cancer as the major reason for worldwide cancer incidence in 2020 (1). Among them, approximately 15% to 25% overexpress HER2 receptors and represent destructive and invasive characteristics and weak consequences (2). Herceptin, one of the current and verified drugs for the therapy of HER2-positive breast cancer, inhibits the ligand-binding region of this kind of tyrosine kinase receptors (3). Unfortunately, one year after treatment with this drug, inherited and acquired resistance to it develops (4). Therefore, the use of drugs that inhibit the tyrosine kinase activity of these receptors such as lapatinib, neratinib, and tucatinib has been increased (5). On the other hand, several pieces of evidence regarding acquired resistance against these drugs have been reported (6). Today, the use of combination chemotherapy with emphasis on the use of phytochemicals with cancer prevention has been further appealing. Such therapy approaches try to use drugs that have less toxic effects, wanted effectiveness, and can be taken by mouth, and are inexpensive (7).

Lapatinib is a commercial drug with potent tyrosine kinase inhibitory properties that specifically inhibits HER2 and EGFR receptors and is still used in clinical trials today. In patients with breast carcinoma who are pretreated with other anti-cancer drugs, lapatinib is usually utilized with other chemotherapeutic agents such as capecitabine (8, 9). It has been reported that lapatinib, in addition to inducing apoptosis in HER2-positive breast cancer cells, also increased the sensitivity of these cells to radiation and restored tamoxifen sensitivity in tamoxifen-resistant breast cancer models (10). 

Metformin is one of the most common insulin-sensitizing drugs used to treat type 2 diabetes (11, 12). Several studies have shown that metformin can decrease the incidence of some cancers, such as breast, colorectal, and liver carcinoma (13-16). Metformin has also been shown to not only inhibits the growth of breast cancer cells but also increases their apoptotic death (17). Recently, combination chemotherapy consisting of chemotherapeutic agents and phytochemicals has received much consideration as a beneficial substitute treatment to improve the effectiveness and reduce the systemic toxic effects of chemotherapeutic agents (18). While Met and Lap have been shown to have anticancer activities, their combination may exhibit a more potent effect against breast cancer. Therefore, we co-treated the human breast-originated carcinoma cell line, SK-BR3, with Met and Lap to investigate the more potent anticancer efficacy of the combination chemotherapy.

## Materials and Methods

***Determination of SK-BR3 cell viability***

The human breast carcinoma cell line, SK-BR3, was obtained from the Pasteur Institute of Iran (Tehran, Iran). Cell viability was determined using an MTT assay. For achieving 60–70% confluency, the SK-BR3 cells were seeded in 6 plates (2 × 10^6^ cells/ well) and incubated at 37 °C. After treatment with metformin and lapatinib alone, and combined for 24, 48, and 72 hr, the media was changed with fresh culture media consisting of MTT solution (0.5 mg/ml), and then, the cells were incubated for a further 4 hr. After dissolving the MTT metabolites, a microplate reader was used to measure the absorbance. Graph Pad Prism program was used to calculate the fifty percent inhibition (IC_50_) of each compound. CompuSyn software was used to evaluate the synergistic relationship between metformin and lapatinib and measure the combination index (CI) based on Chou *et al*. (19). According to this method, CI<1.0, CI>1.0, and CI=1.0 are represented as synergistic, antagonistic, and additive effects, respectively

***Apoptosis assay***

The apoptotic effect of metformin and lapatinib individually and in combination was determined using a commercially available apoptotic kit. After centrifugation of the trypsinized cells, the pellets were washed twice in ice-cold PBS. Then, the cells were re-suspended in a binding buffer containing Annexin V and PI and protected for 10 min in the dark, and counted using a FACS caliber flow cytometer (BD Biosciences, a Jose, CA, USA). Finally, the cell percentage in each quadrant was determined. 

***RNA extraction and quantitative real-time polymerase chain reaction (qRT-PCR)***

Nearly 5 × 10^6^ treated SK-BR3 cells were pelleted, after collecting at subconfluency, entire RNA was obtained with a commercially available kit (Qiagen, Germany). The quantity and integrity of extracted RNA were determined spectrophotometrically and agarose gel electrophoresis, respectively. For c.DNA synthesis 1 μg of extracted RNA was subjected to real-time PCR. cDNA amplification was performed using the following primer sets: 

Bax (5’- GGGTGGTTGGGTGAGACTC-3’, 5’-AGACACGT

AAGGAAAACGCATTA-3); Bcl_2_(5′-TCGCCCTGTGGATG 

ACTGA-3′, 5′-CAGAGACAGCCAGGAG AAATCA-3′); and β-Actin (5’- TGGACTTCGAGCAAGAGATG -3’, 5’-GAAGGAAGGCT GGAAGAGTG -3’). The comparative-Ct method (ΔΔCt method) was used to determine the relative expression of target genes and normalized relative to the expression of the β-actin housekeeping gene. 

***Determination of caspase activity***

A commercially available ELISA kit was used to measure the activity of caspases 3 and 9. Briefly, in a 96-well plate, the cultivated cells were given various concentrations of Met, Lap alone, and combined form. The cells were harvested by trypsinization and they were then pelleted with centrifugation at 1000 ×g for 15 min at room temperature. After that, the other steps were performed based on the manufacturers’ instructions, and absorbance was measured at 570 nm using a plate reader.

***Protein extraction and Western blot analysis***

After treatment of the SK-BR3 cells with Met and Lap alone and combined, the cells were incubated in a lysis buffer containing protease inhibitor cocktail for 30 min on ice. The cell lysates were collected from treated and untreated cells and then used in immunoblotting for determination of AMPK, Akt, and β-actin protein levels. The protein concentration was measured using a commercially available kit (Abcam, Cambridge, UK). After separation of the proteins onto 10% SDS-PAGE gels, they were electrotransferred to PVDF membranes. After transfer, the membranes were blocked with 5% skim milk in TBS with 0.01% Tween-20 (TBS/T) for 15 min. The membranes were then treated with primary anti-Akt (4691s) and anti-AMPK (2532s) antibody, and re-treated with β-actin (4970L) as a housekeeping gene. After washing 3 times for 10 min in TBS/T buffer, the membrane was exposed to an HRP-linked anti-rabbit secondary antibody (7074s; 1/2000 dilution). Finally, the blots were assessed using Bio-Rad ChemiDocTM (Hercules, CA, USA) and quantified with a densitometer. 

***Statistical analysis***

In our study, the quantitative data are reported as mean ± SEM. The MTT assay was used to determine the toxic effects of drugs on cell viability. Graph Pad Prism was used to calculate the IC_50 _of each drug. A one-way analysis of variance (ANOVA) was used to compare the mean between groups followed by a *post hoc* test. *P*<0.05 was considered statistically significant.

## Results

***The synergistic anti-proliferative effects of Met-Lap combination on SK-BR3 cells***

**Figure 1:**
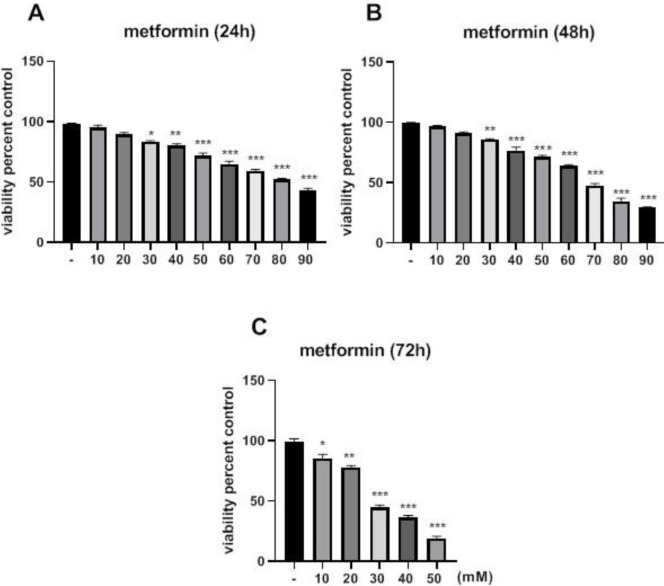
Toxic effect of metformin (Met) on SK-BR3 viability. The cells were incubated with various doses of metformin for 24, 48, and 72 hr

**Figure 2:**
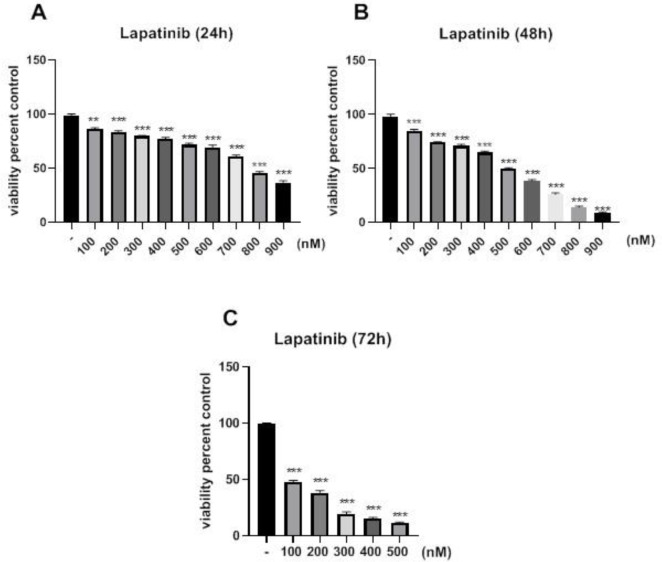
Cytotoxic effect of lapatinib on viability of SK-BR3 cells. The cells were incubated with different concentrations of metformin for 24, 48, and 72 hr, respectively. The MTT assay was used to determine cell viability. Each experiment was performed in triplicate and data were given as means ± SEM. *P<0.05, **P<0.01, and ***P<0.001 vs control

**Figure 3:**
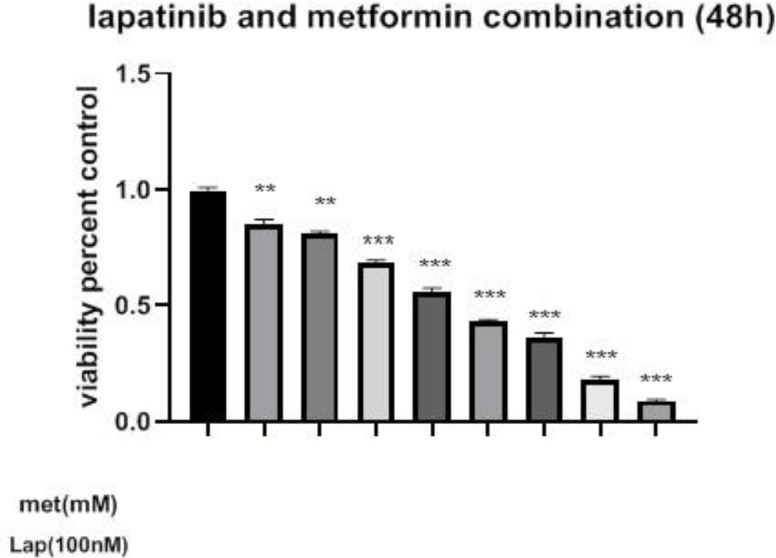
Cytotoxic effect of the metformin-lapatinib combination on the viability of SK-BR3 cells. The cells were incubated with various concentrations of the combination for 48 hr. The MTT assay was used to determine cell viability. Each experiment was performed three times and data were given as means ± SEM. *P<0.05, **P<0.01, and ***P<0.001 vs control

Treatment of the cells with 20 mM of metformin for 24 hr significantly inhibited cell growth versus control (*P*<0.05). When the cells were treated at 48 and 72 hr, their IC_50_ values were decreased to 65 and 25 mM, respectively (Figure 1). Additionally, treatment of the cells with 100 nM of lapatinib for 24 hr significantly inhibited cell growth versus control (*P*<0.05). When the cells were treated at 48 and 72 hr, their IC_50_ values were decreased to 500 and 100 nM, respectively, compared to 800 nM at 24 hr (Figure 2). To determine the synergistic relationship between Met and Lap, the effect of this combination was investigated on SK-BR3 viability. As shown in Figure 3, nearly all Met-Lap combined forms had a potent toxic effect on cell growth versus Lap alone. Additionally, treatment of the cell with 100 mM of metformin combined with 100 nM of lapatinib for 48 hr induced 95% inhibition versus lapatinib alone which displays 15% inhibition. Then we used CompuSyn software to investigate the possibility of the synergistic relationship between metformin and lapatinib. Our results showed that all metformin–lapatinib combinations had a synergistic inhibitory effect on cell viability. However, the potent synergistic inhibitory effects (CI= 0.51) were observed in the concentration of 100 mM metformin plus 100 nM of lapatinib.

***Synergistic apoptotic effects of Met-Lap combination on SK-BR3 cells***

**Figure 4:**
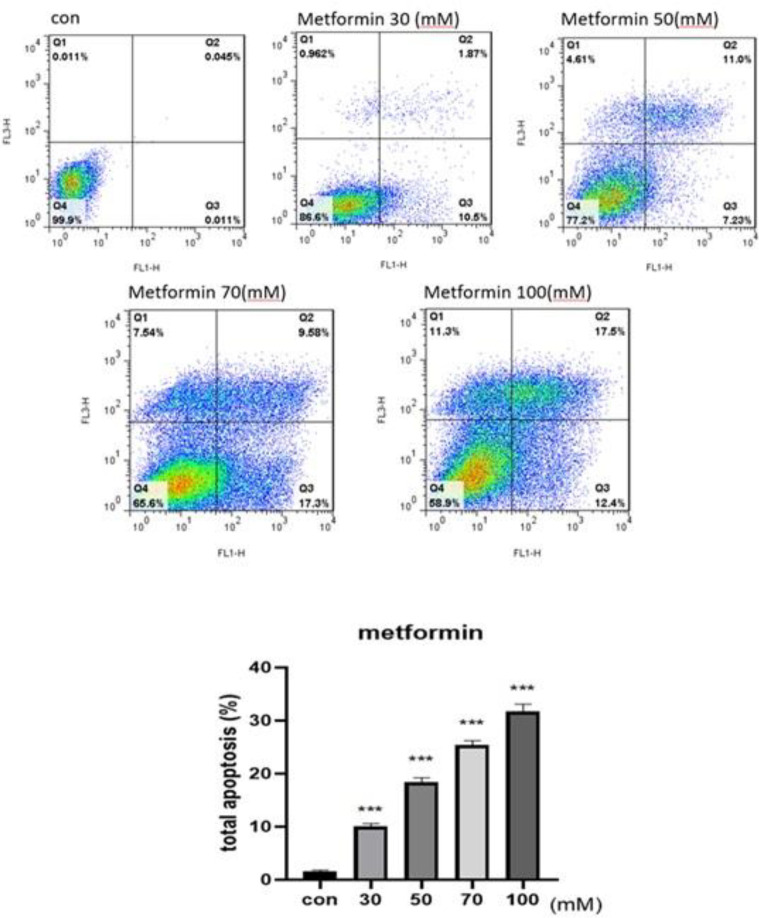
Flow cytometry analysis of metformin-induced apoptotic death in SK-BR3 cells. The cells were incubated with various doses of metformin for 48 hr and then the percentage of apoptotic death was measured. Data are given as means ± SEM. *P*<0.001 vs control

**Figure 5:**
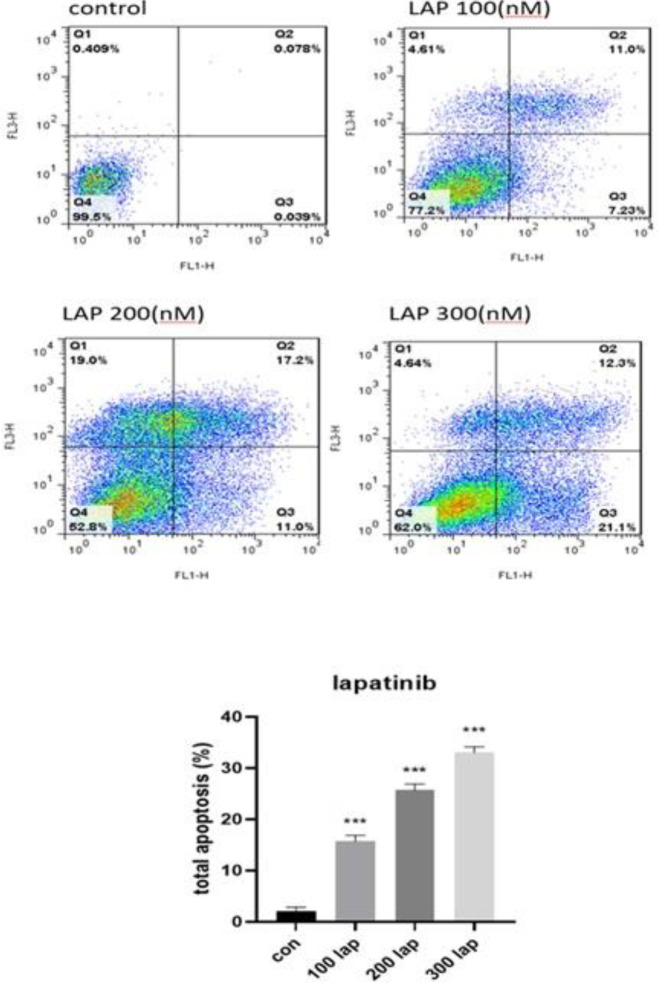
Flow cytometry analysis of lapatinib-induced apoptotic death in SK-BR3 cells. The cells were incubated with various doses of lapatinib for 48 hr, and then the percentage of apoptotic death was measured. Data are given as means ± SEM. ***P<0.001 vs control

**Figure 6:**
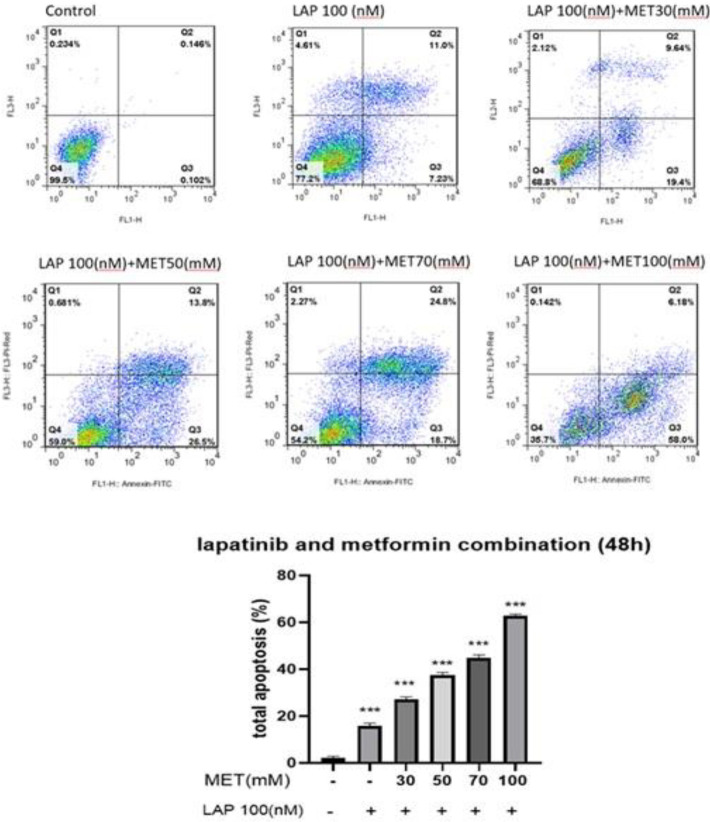
Flow cytometry analysis of the Met-Lap combination-induced apoptotic death in SK-BR3 cells. The cells were incubated with various doses of the metformin-lapatinib combination for 48 hr, and then the percentage of apoptotic death was measured. Lane 1 shows controls; Lane 2 shows the cells given lapatinib alone; lanes 3, 4, and 5 show the cells given the metformin-lapatinib combination. Data are given as means ± SEM. ***P<0.001 vs control

We determined the metformin-lapatinib combination induced apoptotic death using an Annexin V/Propidium iodide staining assay. Our findings indicated that both metformin and lapatinib alone and combined form showed a dose-dependent influence on programmed cell death of SK-BR3. As shown in Figure 4 the calculated cell death was considerably enhanced in treated cells with metformin and lapatinib versus controls (Figure 5). On the other hand, in the cells treated with Lap-Met combination, the calculated cell death was considerably enhanced versus Lap alone (Figure 6). Thus, the Met-Lap combined form could notably enhance the percentage of calculated cell death versus Lap alone.

***Efficacy of Met-Lap combination on the mRNA expression of apoptotic biomarkers in SK-BR3 cells***

**Figure 7:**
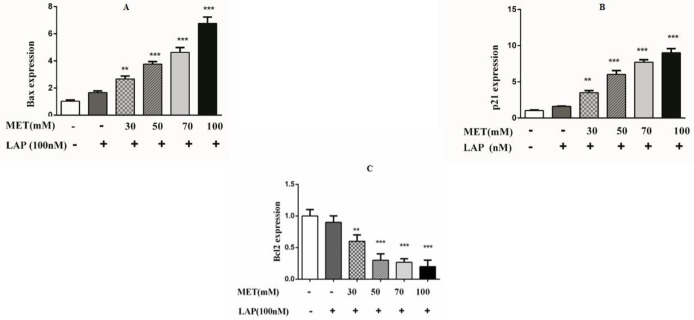
Effect of Met-Lap combination on Bax, P21, and Bcl2 expression in SK-BR3 cells. The cells were incubated with metformin-lapatinib combination for 24 hr, and then the expression of Bax, P21, and Bcl2 was analyzed by qRT-PCR. Data are given as means ± SEM. **P<0.01 and ***P<0.001 vs control

**Figure 8:**
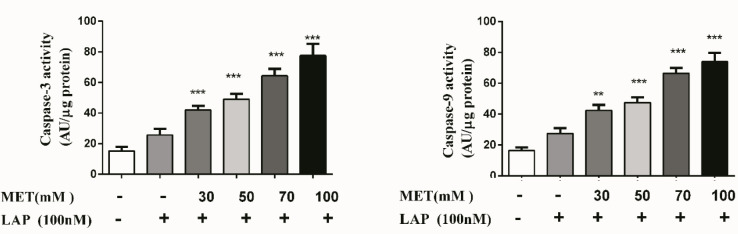
Effect of Met-Lap combination on activity of caspases 3 and 9 in SK-BR3 cells. The cells were incubated with the metformin-lapatinib combination for 24 hr, and then the activity of caspases 3 and 9 was determined using an ELISA kit. Data are given as means ± SEM. **P<0.01 and ***P<0.001 vs control

The data obtained from real-time PCR analysis indicated that the Met-Lap combination significantly increased the expression of Bax and p21 (seven-times and nine-times, correspondingly) (Figures 7A and B), while the expression of Bcl_2_ was considerably reduced by five times at the same drug doses (Figure 7C). These observed data regarding the expressions of cell death markers were according to the results observed in apoptosis analysis. To further confirm the apoptotic influence of the Met-Lap combination, the caspases activity including caspases 3 and 9 were also measured. The obtained data showed that the combination could significantly increase both caspase’s activity. This result is consistent with the consequence of apoptotic data and the expression both of Bax and Bcl_2_ (Figure 8).

***Efficacy of Met- Lap combination on the expression of AMPK and Akt in SK-BR3 cells***

**Figure 9:**
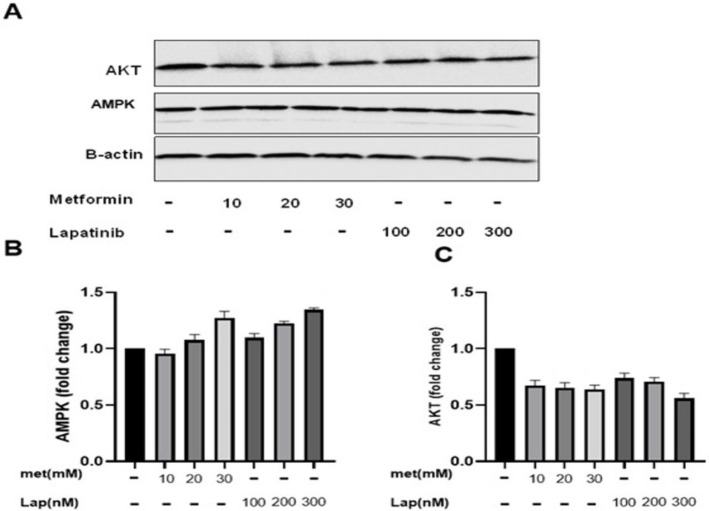
Effect of metformin and lapatinib on the expression of AMPK and Akt in SK-BR3 cells. The cells were incubated with metformin and lapatinib for 48 hr, and then the expression of AMPK, Akt, and β-actin was analyzed by Western blotting. Data are given as means ± SEM. *P<0.05 and **P<0.01 vs control

**Figure 10:**
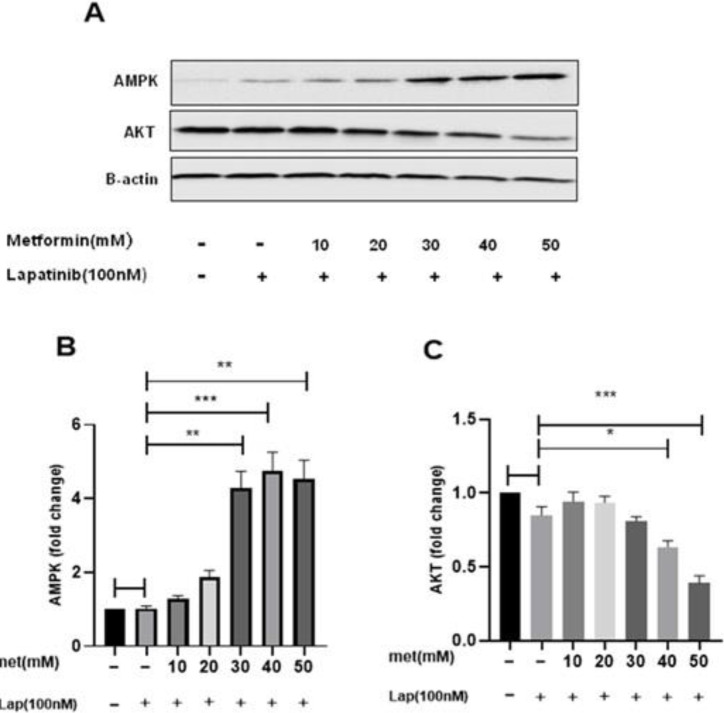
The effect of the metformin-lapatinib combination on the AMPK and Akt expression in SK-BR3 cells. The cells were treated with the Met-Lap combined form at 48 hr and then the expression of AMPK, AKT, and β-actin was analyzed by Western blotting. Data are given as means ± SEM. *P<0.05, **P<0.01, and ***P<0.001 vs control

To assess the mechanism of metformin and lapatinib alone and in combination in programmed cell death, the expression of AMPK and Akt was also measured. Western blot analysis indicated that Met and Lap alone didn’t considerably change Akt and AMPK expression (Figure 9). Whereas, after incubation of the cells with the combination at 48 hr, Akt expression was considerably reduced, and AMPK expression was considerably enhanced versus both of them alone (Figure 10).

## Discussion

 Alterations in HER2 function are some of the most common molecular abnormalities in breast carcinoma and are generally related to destructive phenotype and weak consequences (2, 3). Practically, two major limitations have been arising following the use of HER2 receptor blockers in patients with overexpressing of this receptor. The first limitation is that their effect is usually limited one year after taking them due to inherited or acquired resistance (4). The second limitation is that due to the important role of HERE2 in the activity and development of the heart, cardiomyopathy should be considered (20, 21). Therefore, it has been suggested that inhibition of HER2 along with AMPK activation may reduce cardiac complications and increase the success of cancer treatment (22). Numerous studies have also shown that combination chemotherapy has great potential in reducing malignant cell growth, inhibiting angiogenesis, inducing apoptosis, and stimulating the immune system (8-10). Nowadays, combination chemotherapy consisting of chemotherapeutic agents and phytochemicals has received a lot of consideration as a beneficial substitute treatment to enhance the effectiveness and reduce the systemic impact of medications. Even though several reports exist about the effect of metformin in combination with other drugs on the reduction of cell growth and programmed cell death, the mechanism of action of the metformin-lapatinib combination should be more investigate and clarified. Therefore, in the present study, we investigated the effect of metformin and lapatinib alone and in combined form on cell survival and apoptosis in SK-BR3 cells.

Our results indicated that metformin and lapatinib alone and in combined form have time and dose-dependent considerable toxic effects on the viability of SK-BR3 cells. The results of flow cytometry data showed that treatment with the metformin-lapatinib combination has stronger apoptotic effects versus treatment alone. Additionally, the Met-Lap combined form enhanced the expression of Bax as a pre-apoptotic protein and P21 as an inhibitory marker and both caspases activity, while it reduced the mRNA expression of Bcl_2_ as an anti-apoptotic factor. These findings are consistent with the results of the previous studies regarding the toxic and inhibitory effects of metformin (17, 23, 24) and lapatinib (10, 25, 26) on the growth of breast cancer cells. 

In the clinic, metformin is a safe, readily available, and inexpensive drug. Although metformin is the first oral anti-diabetic drug, several studies have shown its anti-cancer effects on several malignancies, including the pancreas, prostate, colon, and breast. (14-17). Several mechanisms have been proposed, including induction of AMPK and reduction of the mTOR signaling pathway (15,27) to explain the anti-cancer effects of metformin. It has already been shown that because Akt is downstream of various growing pathways, it has a crucial role in the cell death and proliferation of tumor cells (28,29). Therefore, our finding that the metformin-lapatinib combination can reduce Akt expression indicates its key role in the regulation of cancer cells. Jiralerspong *et al*. (30) in a clinical trial study showed that diabetic patients with breast cancer had a better pathological response if they took metformin simultaneously with chemotherapy than those who did not. Oliveira-Ferraro *et al*. (31) in a study in breast cancer cells showed that metformin, by activating AMPK, could inhibit mitosis phase-related genes, and thus can arrest the cells in the M phase. In another study (14), they showed that metformin prevents the proliferation of prostate cancer cells but did not affect cell death. On the other hand, other researches have shown metformin increased cell death in breast, colon, and endometrial cancers (32-34). It has already been demonstrated that caspases have an important role in the initiation and progression of apoptosis. Among them, caspase 3 has a vital role in the execution phase of the apoptosis pathway. Our findings showed metformin and lapatinib individually and in combined form increased both caspases activity in treated cells, so their effect on the induction of apoptosis can be mediated via a caspase-dependent pathway. A study (34), showed that metformin enhanced cell death in all breast carcinoma cells by increasing the activity of caspase and PARP, and so, these results are consistent with our findings.

Lapatinib, a dual tyrosine kinase inhibitor of the HER2 receptors, is clinically used in HER2 positive breast cancer patients, but the mechanisms of its antitumor effects have not yet been determined. On the other hand, the Akt / PKB pathway is an important kinase that not only has a key role in the modulating of several intracellular pathways but also in glucose metabolism and angiogenesis (17). Western blot data showed both metformin and lapatinib alone did not alter the AMPK and Akt expression, but their combined form induced up-regulation of AMPK and down-regulation of Akt expression. Previous studies have shown that metformin can inhibit several intracellular signaling transductions through two key proteins ERK and Akt, which have an important role in the pathogenesis of several malignancies, including breast cancer (35, 36). It has been shown that dysregulation of the Akt signaling pathway occurs in different types of malignancies (36). Modulation of cell death by Akt may also be achieved by controlling the phosphorylation of numerous pro-apoptotic factors, such as Bax and caspase-9 (37). We have observed that metformin and lapatinib alone and in combined form can increase the Bax expression and both caspase’s activity. It is thought that these effects may be due to a decrease in Akt levels. Akt is also involved in controlling the expression of several pro-apoptotic genes through phosphorylation and inhibition of several transcription factors. On the other hand, Akt can reduce the expression of several anti-apoptotic genes by phosphorylation and inactivation of the IκB kinase (IKK) (38). Additionally, Akt can reduce the mRNA expression of P53 by phosphorylation of several transcription factors (39). Our results showed metformin and lapatinib alone and in combined form enhance the expression of P21, as one of the P53 targets. Therefore, we suggest that this observed effect probably occurs through the elimination of the Akt inhibitory effect on P53 expression.

The 3 (4,5 Dimethyl 2 Thiazolyl) 2,5 Diphenyltetrazolium Bromide (MTT) assay was used to determine cell viability. Each experiment was performed in triplicate and data were given as means ± SEM. *P<0.05, **P<0.01, and ***P<0.001 vs control

**Table 1:** Combination index (CI) calculated between metformin (Met) and lapatinib (Lap)

**MET** **(100 mM)**	**MET** **(70 mM** **)**	**MET** **(50 mM)**	**MET** **(40 mM)**	**MET** **(30 mM** **)**	**MET** **(20 mM)**	**MET** **(10 mM)**	
**0.51**	**0.59**	**0.72**	**0.73**	**0.79**	**0.82**	**0.89**	**L** **AP** **(100 nM)**

SK-BR3 cells incubated with 100 nM of lapatinib and various concentrations of metformin. CI was calculated by CompuSyn software. The synergistic, additive, and antagonistic effects are expressed as CI<1, =1, and >1, correspondingly

## Conclusion

Metformin can synergistically increase Lapatenib’s toxic effects on cell viability and its induced programmed cell death by up-regulation of apoptotic factor expression including Bax and Bcl_2_ and by increasing caspases 3 and 9 activity in SK-BR3 cells. These synergistic effects of the Met-Lap combined form may be mediated by up-regulating and down-regulating of AMPK, and Akt expression, respectively. Our findings also confirm the results of the previous studies that showed metformin can make cancer cells more sensitive to chemotherapy drugs and provide valid evidence of the therapeutic potential of metformin for intervention in patients with breast carcinoma that overexpress HER2 receptors.

## Authors’ Contributions

GM study design, draft manuscript preparation, and critical revision of the paper; AH and MA supervision of the research; AK flow cytometry and Western blot analysis; DN and KAB performing experiments, analysis, and interpretation of the results; MM data analysis and manuscript revision; GM, AH, MA, AK, DN, KAB, and MM final approval of the version to be published.

## Conflicts of Interest

The authors declare no conflicts of interest and are fully responsible for data collection, originality, interpretation, and writing of this manuscript.

## References

[1] Sung H, Ferlay J, Siege RL, Laversanne M, Soerjomataram I, Jemal A (2021). Global cancer statistics 2020: GLOBOCAN estimates of incidence and mortality worldwide for 36 cancers in 185 countries. CA Cancer J Clin.

[2] Li S, Wu J, Huang O, He J, Zhu L, Chen W (2020). HER2 positivity is not associated with adverse prognosis in high-risk estrogen receptor-positive early breast cancer patients treated with chemotherapy and trastuzumab. Breast.

[3] Jagosky M, Tan AR (2021). Combination of pertuzumab and trastuzumab in the treatment of HER2-positive early breast cancer: a review of the emerging clinical data. Breast Cancer (Dove Med Press).

[4] Gandullo-Sánchez L, Capone E, Ocaña A, Iacobelli S, Sala G, Pandiella A (2020). HER3 targeting with an antibody-drug conjugate bypasses resistance to anti-HER2 therapies. EMBO Mol Med.

[5] Yang X, Wu D, MD, Yuan S (2020). Tyrosine kinase inhibitors in the combination therapy of HER2 positive breast cancer. Technol Cancer Res Treat.

[6] Rosenzweig SA (2018). Acquired resistance to drugs targeting tyrosine kinases. Adv Cancer Res.

[7] Mitra S, Dash R (2018). Natural products for the management and prevention of breast cancer. Evid Based Complement Alternat Med.

[8] Ma F, Ouyang Q, Li W, Jiang Z, Tong Z, Liu Y (2019). Pyrotinib or Lapatinib Combined with Capecitabine in HER2-Positive Metastatic Breast Cancer with Prior Taxanes, Anthracyclines, and/or Trastuzumab: A Randomized, Phase II Study. J Clin Oncol.

[9] Saura C, Oliveira M, Feng YH, Dai MS, Chen SW, Hurvitz SA (2020). Neratinib plus capecitabine versus lapatinib plus capecitabine in her2-positive metastatic breast cancer previously treated with 2 HER2-directed regimens: phase III NALA trial. J Clin Oncol.

[10] Nahta R, Yuan LX, Du Y, Esteva FJ (2007). Lapatinib induces apoptosis in trastuzumab-resistant breast cancer cells: effects on insulin-like growth factor I signaling. Mol Cancer Ther.

[11] Zhou T, Xu X, Du M, Zhao T, Wang J (2018). A preclinical overview of metformin for the treatment of type 2 diabetes. Biomed Pharmacother.

[12] LaMoia TE, Shulman GI (2021). Cellular and molecular mechanisms of metformin action. Endocr Rev.

[13] Kamarudin MNA, Sarker MMR, Zhou JR, Parhar I (2019). Metformin in colorectal cancer: molecular mechanism, preclinical and clinical aspects. J Exp Clin Cancer Res.

[14] Sahra IB, Laurent K, Loubat A, Giorgetti-Peraldi S, Colosetti P, Auberger P (2008). The antidiabetic drug metformin exerts an antitumoral effect in vitro and in vivo through a decrease of cyclin D1 level. Oncogene.

[15] Cunhaa V, Cotrima HP, Rochab R, Carvalhoa K, Lins-Kusterer L (2020). Metformin in the prevention of hepatocellular carcinoma in diabetic patients: A systematic review. Ann Hepatol.

[16] Vancura A, Bu P, Bhagwat M, Zeng J, Vancurova I (2018). Metformin as an anticancer agent. Trends Pharmacol Sci..

[17] Shi P, Liu W, Wang H, Li F, Zhang H, Wu Y (2017). metformin suppresses triple-negative breast cancer stem cells by targeting KLF5 for degradation. Cell Discov.

[18] Rizeq B, Gupta I, Ilesanmi J, AlSafran M, Rahman M, Ouhtit A (2020). The power of phytochemicals combination in cancer chemoprevention. J Cancer.

[19] Chou TC (2006). Theoretical basis, experimental design, and computerized simulation of synergism and antagonism in drug combination studies. Pharmacol Rev.

[20] Copeland-Halperin RS, Liu JE, Yub AF (2019). Cardiotoxicity of HER2-targeted therapies. Curr Opin Cardiol.

[21] Jafari L, Akhter N (2021). Heart failure prevention and monitoring strategies in HER2-positive breast cancer: a narrative review. Breast Cancer Res Treat.

[22] Jhaveri TZ, Woo J, Shang X, Park BH, Gabrielson E (2015). AMP-activated kinase (AMPK) regulates activity of HER2 and EGFR in breast cancer. Oncotarget.

[23] Jafari-Gharabaghlou D, Pilehvar-Soltanahmadi Y, Dadashpour M, Mota A (2018). Combination of metformin and phenformin synergistically inhibits proliferation and hTERT expression in human breast cancer cells. Iran J Basic Med Sci.

[24] Besli N, Yenmis G, Tunçdemir M, Sarac EY, Doğan S, Solakoğlu S (2020). Metformin suppresses the proliferation and invasion through NF-kB and MMPs in MCF-7 cell line. Turk J Biochem.

[25] Abo-Zeid MAM, Abo-Elfadl MT, Gamal-Eldeen AM (2019). Evaluation of lapatinib cytotoxicity and genotoxicity on MDA-MB-231 breast cancer cell line. Environ Toxicol Pharmacol.

[26] Guan M, Tong Y, Guan M, Liu X, Wang M, Niu R (2018). Lapatinib Inhibits Breast Cancer Cell Proliferation by Influencing PKM2 Expression. Technol Cancer Res Treat.

[27] Guo L, Cui J, Wang H, Medina R, Zhang S, Zhang X (2021). Metformin enhances anti-cancer effects of cisplatin in meningioma through AMPK-mTOR signaling pathways. Mol Ther Oncolytics.

[28] Ianza A, Sirico M, Bernocchi O, Generali D (2021). Role of the IGF-1 axis in overcoming resistance in breast cancer. Front Cell Dev Biol.

[29] Jiang N, Dai O, Su X, Fu J, Feng X, Peng J (2020). Role of PI3K/AKT pathway in cancer: the framework of malignant behavior. Mol Biol Rep.

[30] Jiralerspong S, Palla SL, Giordano SH, Meric-Bernstam F, Liedtke C, Barnett CM (2009). Metformin and pathologic complete responses to neoadjuvant chemotherapy in diabetic patients with breast cancer. J Clin Oncol.

[31] Oliveira-Ferraros C, Vazquez-Martin A, Menendez JA (2009). Genome-wide inhibitory impact of the AMPK activator metformin on kinesins, tubulins, histones, auroras and polo like kinases M-phase cell cycle genes in human breast cancer cells. Cell Cycle.

[32] Berstein LM, Yue W, Wang JP, Santen RJ (2010). Isolated and combined action of tamoxifen and metformin in wild-type, tamoxifen-resistant, and estrogen deprived SKBR3cells. Breast Cancer Res Treat.

[33] Topcul M, Cetin I (2015). Effects of metformin on cell kinetic parameters of MCF-7 breast cancer cells in vitro. Asian Pac J Cancer Prev.

[34] Zhuang Y, Miskimins WK (2011). Metformin induces both caspase-dependent and poly(ADP-ribose) polymerase-dependent cell death in breast cancer cells. Mol Cancer Res.

[35] Mundi PS, Sachdev J, McCourt C, Kalinsky K (2016). AKT in cancer: new molecular insights and advances in drug development. Br J Clin Pharmacol.

[36] Steelman LS, Navolanic P, Chappell WH, Abrams SL, Wong EW, Martelli AM (2011). Involvement of AKT and mTOR in chemotherapeutic- and hormonal-based drug resistance and response to radiation in breast cancer cells. Cell Cycle.

[37] Nitulescu GM, Van De Venter M, Nitulescu G, Ungurianu A, Juzenas P, Peng Q (2018). The Akt pathway in oncology therapy and beyond (Review). Int J Oncol.

[38] Tang B, Tang F, Wang Z, Qi G, Liang X, Li B (2016). Upregulation of Akt/NF-κB-regulated inflammation and Akt/Bad-related apoptosis signaling pathway involved in hepatic carcinoma process: suppression by carnosic acid nanoparticle. Int J Nanomedicine..

[39] Park S, Kim D, Dan HC, Chen H, Testa JR, Cheng JQ (2016). Identification of an AKT interaction protein, PHF20/TZP, that transcriptionally regulates p53. J Biol Chem.

